# Supplementary material for: MetaRibo-Seq measures translation in microbiomes
Source: Nat Commun. 2020 Jun 29;11:3268. doi: 10.1038/s41467-020-17081-z (PMC7324362; doi:10.1038/s41467-020-17081-z)
Supplement: Supplementary file 10 — Supplementary Data 7 [file 41467_2020_17081_MOESM10_ESM.zip › File2/Confidence_High_Taxonomy/230074_out.krona.html]

Javascript must be enabled to view this page.

members
magnitude
magnitudeUnassigned
count
unassigned
taxon
rank

230074\_out

2

2
2
superkingdom

2
1239
phylum

186801
2
class

2
186802
order

1
186803
family

1
841
genus

species

SRS048060\_contig\_number\_2226
2049040
1

family
186806
1

genus
1730
1

142586

SRS148159\_contig\_number\_43951
1
species
